# Disruption of the thyroid hormone system and patterns of altered thyroid hormone serum concentrations after chemical exposures in adult rodents – a systematic review

**DOI:** 10.3389/fendo.2026.1911254

**Published:** 2026-09-28

**Authors:** Asma H. Baig, Isabel Forner-Piquer, Sibylle Ermler, Andreas Kortenkamp

**Affiliations:** 1 Centre for Pollution Research and Policy, College of Health, Medicine and Life Sciences, Brunel University of London, Uxbridge, United Kingdom; 2 Institute of Marine Science, Spanish National Research Council (ICM-CSIC), Passeig Maritim de la Barceloneta, Barcelona, Spain; 3 Centre of Genome Engineering and Maintenance, Department of Biosciences, College of Health, Medicine and Life Sciences, Brunel University of London, Uxbridge, United Kingdom

**Keywords:** endocrine disrupting chemicals, systematic review, thyroid hormone system disruption, thyroid stimulating hormone (TSH), thyroxine (T4)

## Abstract

**Introduction:**

Identification of thyroid hormone system-disrupting chemicals (THSDC) requires a solid understanding of the mechanisms by which exposure leads to adverse outcomes in the thyroid hormone system (THS).

**Methods:**

In this systematic review, we present the results from our extensive evidence mapping of thyroid hormone (TH) and thyroid stimulating hormone (TSH) patterns in serum or plasma, after exposure to various THSDCs in adult rodents. Following PRISMA guidelines, we systematically searched PubMed, Web of Sciences and Scopus for relevant studies. Eligible studies were selected according to predefined inclusion and exclusion criteria.

**Results:**

Our review of 234 peer-reviewed studies revealed that only a small group of compounds induced changes in T4 and TSH consistent with the canonical view of the hypothalamic-pituitary-thyroid (HPT) negative feedback loop, whilst the most frequent pattern was a decrease in T4 without TSH changes. The lack of a TSH response may have been due to T4 decreases not being sufficient to change HPT feedback. However, risk of bias analysis also identified deficits in TH/TSH analytical methodology, including the timepoints of TH measurements, possibly explaining discrepancies between studies. We further compared T4/TSH patterns to mechanism-of-action information and other THS-related outcomes and found that THSDCs frequently affected TH synthesis, induced hepatic enzymes involved in TH metabolism, and exhibited other differences in pharmacokinetic properties. Given the diverse chemical nature of the various THSDCs and variety of molecular targets in the THS, there is likely no single explanation for the different patterns observed.

**Conclusion:**

Our compilation of T4/TSH patterns observed after different chemical exposures demonstrated that it is important to not solely rely on the canonical serum hormonal patterns to determine adversity of chemical exposures. Chemicals with different responses should be carefully considered, as current regulatory criteria may fail to identify them as being harmful to human health.

**Systematic review registration:**

https://zenodo.org/records/5528557, identifier doi.org/10.5281/zenodo.5528557.

## 1 Introduction

Accumulating evidence from epidemiological and animal studies highlights the crucial role of maintaining thyroid hormone (TH) balance in the development and maintenance of many physiological processes. Concentrations of thyroxine (T4), the primary product of the thyroid gland and TSH, a pituitary hormone which stimulates the thyroid gland to produce T4, are tightly regulated by the hypothalamic-pituitary-thyroid (HPT)-axis via a negative feedback loop (1). T4 is released from the thyroid gland into the blood stream and converted at tissue level into the biologically active form triiodothyronine (T3). Both hormones can be measured in their free forms or bound to distributor proteins in the blood (2). While most of the hormones are bound to binding proteins, only the free fraction remains active and available. When T3 and T4 reach their target site in the tissue, they dissociate from the binding protein and can enter cells via TH transporters. T4 is converted to T3 by cytoplasmic deiodinases, and T3 then binds to nuclear TH receptors at DNA response elements, activating transcription and driving cell-specific gene expression (3, 4).

Multiple components of the thyroid hormone system (THS) are directly or indirectly involved in the fine-tuning of TH levels through peripheral and central regulation. These include distributor proteins, membrane transporters, deiodinases, enzymes involved in synthesis and metabolism, TH receptor interaction, or modulation of iodide uptake into the thyroid, all of which contribute to HPT-axis regulatory action. This presents many potential targets within the THS that are vulnerable to interference by endocrine disrupting chemicals (EDCs) (5). Although exposures to these compounds are usually very low, they are ubiquitous in the environment, and repeated or chronic exposure can lead to THS disruption (6).

As reviewed previously (7), perchlorate, polybrominated (PBDEs) and polychlorinated (PCBs) compounds or bisphenol A (BPA) are examples of compounds targeting various THS components. Their targets include the sodium-iodide symporter (NIS), the binding protein transthyretin (TTR), the membrane transporter monocarboxylate transporter 8 (MCT8) and the metabolizing sulfotransferases (SULT) enzymes, resulting in an imbalance of TH homeostasis.

One indicator for THS imbalance, which is readily accessible in both human clinical and animal experimental settings, is a decrease in T4 levels measured in serum, leading to a hypothyroid state and resulting in reduced negative feedback inhibition, causing an increase in TSH levels (8). Chronically high serum levels of TSH can cause thyroid hyperplasia and hypertrophy, leading to increased thyroid weight, which may be implicated in thyroid gland carcinogenesis (9–13). Both T4 decrements and changes in thyroid histopathology in animal studies are the current criteria in EU regulations for identifying THSDCs. Serum hormone profile changes coupled with increased thyroid weight and histopathology are indicative of an endocrine mechanism and meet the European Food Standards Agency (EFSA) criterion for adversity (14).

However, studies have shown that some chemicals which do not induce the full spectrum of decreased T4, increased TSH, and abnormal thyroid histopathology can still adversely affect the developing brain, for instance by impacting neurodevelopment (15–17) which was found to be correlated with decreased T4 levels (18, 19) but not necessarily changes in histopathology (20). Such reports indicate that the canonical view of the HPT-axis-steered regulation of the THS is not always applicable. Neurotoxic effects in the absence of obvious thyroid-related changes may also be due to direct neurotoxicity or involvement of other endocrine systems (21, 22). Although the mechanisms behind these unusual, non-canonical patterns in thyroid-related effects are not yet fully understood, other mechanisms extraneous to the HPT-axis, such as hepatic metabolism, were suggested to be involved (23, 24).

We demonstrated in our previous systematic review that gestational/lactational exposures to different classes of chemicals dysregulated T4/TSH signaling in rodent pups and dams (25). In the present study, we systematically reviewed the peer-reviewed scientific literature, with particular emphasis on direct exposures, and collated studies focusing on the period between post-lactation, i.e. in directly (non-maternal) exposed pups and adolescents up to full rodent adulthood. We extracted data on hormonal levels (free and total TH) and other THS endpoints, together with a confidence rating for each study and included data related to Mechanism of Action (MoA) where available. This systematic review aimed to provide an evidence base for regulatory assessment of chemicals capable of disrupting THS homeostasis, with additional findings related to MoAs. The precise mechanisms by which THSDCs alter serum THs levels remain unresolved, but several possibilities are presented and discussed.

## 2 Materials and methods

### 2.1 Literature search and screening

The systematic review methodology followed COSTER recommendations (26) and is reported in accordance with the Preferred Reporting Item for Systematic Reviews and Meta-Analysis (PRISMA) checklist (27). The detailed protocol for this systematic review can be found in the open-access repository Zenodo, uploaded in 2021 (https://zenodo.org/record/5528557). This initial protocol was written to compile data for humans and laboratory animals in one review. Due to the large number of studies, we decided to report human and rodent results separately, and then to further split adult rodents and offspring (25).

For study selection, we used the PECO statement (Population: Laboratory mammalian species, Exposure: All chemical classes by gavage, drinking water or diet, Comparator: Animals not exposed, Outcome: Thyroid hormone measurements in serum/plasma) to frame the research question, consistent with the methodology applied in our previous publication (**Table 1**) (25).

**Table 1:** PECO statement.

PECO element	Description	Inclusion criteria	Exclusion criteria
Populations	Laboratory mammalian species	Terrestrial mammalian species	Non-mammalian test species such as fish or amphibians. Marine mammals
Exposures	All chemical classes: chemicals – natural and synthetically produced with hormonal activity	Administered by gavage, drinking water or diet, at least 2 dose groups	Administered subcutaneously or intraperitoneally; only 1 exposure dose group
Comparators	Groups with no chemical exposure or exposure to vehicle	Control group (same species as exposure group(s)	No control group
Outcomes	Thyroid hormone measurements in serum/plasma	Serum/plasma concentrations of total and free T4, total and free T3, TSH	No T4 measurements No TSH measurements

The literature search was conducted in three databases: PubMed, Web of Science and Scopus. We employed search terms relevant to the THS and EDCs to capture information on the effects of chemicals on T4/TSH. We only included peer-reviewed journal articles and no grey literature or published regulatory data sets in our searches, to facilitate a systematic search approach and risk of bias (RoB) analysis.

A pilot study in September 2020 was followed by refining the search string using the class names of chemicals. An update was made in July 2021 to modify the search strategy, and to avoid bias capturing only literature with specific chemicals. After another iteration, we built the final search string including material items commonly known to contain potential EDCs and avoiding specific chemical names. The list of items included was compiled following Forner-Piquer et al., 2023 (25). Furthermore, we updated the systematic review in January 2024 to include new studies published between July 2021 and December 2023.

We applied no limits to publication or study date, and publication language was restricted to English. The following final search string was applied, slightly modified depending on the database: ((“Endocrine disrupt*” OR EDC OR “thyroid disrupt*” OR estrogen* OR fungicid* OR insecticid* OR rodenticid* OR bactericid* OR antimicrobial* OR herbicid* OR pesticid* OR biocid* OR cosmetic* OR “personal care product*” OR “UV filter*” OR pharmaceutical* OR “heavy metal*” OR flam* OR mycotoxin* OR microcystin* OR xeno* OR plastic* OR textil* OR clothing* OR furniture OR pack* OR “canned food*” OR bottle OR container OR “medical device*” OR paint* OR adhesiv* OR solvent* OR propellant* OR coating* OR electronic* OR household OR product* OR toy OR dust OR waste) AND (TSH OR thyrotrop* OR thyroid* OR “HPT axis” OR “nuclear hormone receptor”) AND (T4 OR thyroxine* OR tetraiodothyronine OR T3 OR triiodothyronine OR “thyroid hormone*” OR “TH”)). Detailed search strategies and a list of terms are available in Forner-Piquer et al. (25).

The systematic review process was managed using the freely available online tool CADIMA (28). Title and abstract screening followed by full-text screening was conducted by two team members working independently. A consistency check with a subsample of 200 randomly selected studies was performed, which achieved a kappa value of 0.55, considered as “fair” by CADIMA criteria. Any potential conflicts among both screeners during the title/abstract or full-text screening were resolved after discussion or by inclusion of a third reviewer.

From the merged list only peer-reviewed, primary research studies were selected for data extraction and study evaluation. When different studies reported the same data, we selected the study reporting the most complete dataset.

We included all experimental studies (*in vivo*) with rodent models (rat and mouse) and other mammalian species (e.g. guinea pig) that measured serum/plasma levels of T4 (total and/or free) and TSH following chemical administration through the diet, drinking water or gavage. Rodents are considered adults at 6 weeks of age; however, pups or juvenile rodents were included when the test compound was orally administered and not via lactation. We did not include an upper age limit. Studies including other factors (e.g. animals with chronic diseases) were not selected.

We focused on studies that tested individual compounds at multiple doses, which can reveal dose-dependent effects. Although studies with only one dose of test chemicals did not meet our eligibility criteria, their outcomes were considered nonetheless to investigate whether their response patterns were consistent with eligible studies. Studies with non-mammalian test species were excluded as well as studies not following oral administration, and studies with marine mammals.

### 2.2 Data extraction and synthesis

Data from the selected studies were extracted into a Microsoft Excel spreadsheet adapted from the one described in Martin et al. (29). The key data extraction elements to capture metadata, study details on animal model, study design, methods and outcomes are provided in **Table 2**; **Supplementary Material 1**.

**Table 2:** Summary of key data extraction elements.

Type of data	Key elements
Metadata	article title
	authors
	publication year
	journal name
	funding source
	funding source category
Information about the study	Animal model (species, strain, age at the beginning and at the end of the treatment or at the time the TH/TSH were measured).
	Study design (duration, exposure concentration, exposure regimen, dosing route, inclusion of negative and positive control, thyroid histopathology, hepatic T4-UDPGT, measurement method for T4/T3, measurement method for TSH, biological sample measured, statistical methods).
	Chemical characterisation (chemical name, acronym, CAS registry number, chemical class, chemical uses, source of the chemical, purity, vehicle, chemical detection: tissue and method).
	Hormone measurements in adult rodents: TT4, FT4, TT3, FT3, TSH (indicated as: increase “˄”, decrease “˅”, no change “--”).

TH and TSH levels were included as reported in the studies: statistically significantly increased, decreased, or unchanged in relation to control groups (no test compound exposure). To enable comparability across studies we also calculated the percentage change in the treatment groups compared to control groups and to quantify the T4/TSH alterations. Percentage changes of T4, fT4 and TSH were calculated with the following formula: *[((TH level in experimental group – TH level in control group)/ TH level in control group) x 100].* Percentage changes were calculated for the reported means of each experimental group. For TH/TSH data presented in graphs, the freely available online tool WebPlotDigitizer (30) was used to extract the data.

For articles that tested more than one chemical, we recorded one entry for each chemical (or unit of evidence) in the data extraction template.

This study also aimed to examine the relationships between MoA and T4/TSH patterns. Thus, where available, we also extracted data for other endpoints related to the THS and collated T4/TSH patterns with these MoAs. THS-related endpoints included:

- T4-UDP-glucuronosyltransferase (UDPGT) activity, (enzyme involved in hepatic catabolism of TH).
- Thyroid histopathology
- Gene, protein markers or enzymatic activities involved in TH synthesis, TH/TSH regulation, TH transport/binding or hepatic metabolism.
- Other outcomes such as peripheral tissue TH levels, serum thyrotropin-releasing hormone (TRH), iodide uptake or TH clearance.

### 2.3 Study evaluation (risk of bias assessment)

The internal validity of the selected studies was appraised with a risk of bias (RoB) assessment adapted from (25, 31–33) and the National Toxicology Program - Office of Health Assessment and Translation (NTP-OHAT) (34). For publications which assessed several chemicals, we recorded one RoB entry for each chemical (unit of evidence). The RoB tool comprised a list of 18 questions with 4 response options based on NTP-OHAT scores: ++ Definitely low risk of bias (dark green); + Probably low risk of bias (light green); ~ Probably high risk of bias (orange); ~~ Definitely high risk of bias (red) (**Supplementary Material 2**).

Among the 18 questions, we included 8 key questions which had to score at “definitely low risk” or “probably low risk” to rate a study as “high confidence”.

These 8 key elements were:

1. and 2. Reliability and sensitivity of the analytical methods used for serum T4 and TSH quantification, respectively. We scored articles as “~ Probably high risk of bias (orange)” or “~~ Definitely high risk of bias (red)” when: i) details about the methodology or the assays were missing, ii) the authors added the code or the supplier of the kit used, but we were unable to review the characteristics for the analytical measurements, for example, at the supplier website or through the reference cited, iii) there was no mention of TH analytical methods, iv) when the assay used was developed for human serum without any further adaptation/validation for the rodent serum matrix. Accordingly, when the characteristics of the assays or kits were available, with technical details, we scored studies as “++ Definitely low risk of bias (dark green)” or “+ Probably low risk of bias (light green)”.
3. Exposure characterization, in terms of purity of the chemical, route of administration or measurement of real concentrations in the diet or drinking water. Studies with a comprehensive description of the exposure were classified as “++ Definitely low risk of bias (dark green)” or “+ Probably low risk of bias (light green)” when the study included a list of minimal information such as method of administration, concentrations, carrier solvent or duration of the exposure. When those details were missing or if we considered the information regarding the exposure as insufficient, the study was classified as “~ Probably high risk of bias (orange)”. Studies with direct evidence of inconsistent administrations were marked as “~~ Definitely high risk of bias (red)”.
4. Numbers of animals used per dose. Studies using 5 or more animals per experimental group were marked as “++ Definitely low risk of bias (dark green)”, while studies using fewer than 5 animals but with significant effects on TH levels were classified as “+ Probably low risk of bias (light green)”. Studies with insufficient information or direct evidence of using a lower number of animals per group were marked as “~ Probably high risk of bias (orange)” or “~~ Definitely high risk of bias (red)”, respectively.
5. Inclusion of a positive control for producing hormonal changes. Demonstration of effects with a positive control was taken as evidence of a responsive animal model. Accordingly, when a positive control was ineffective in terms of TH/TSH alterations, the study was ranked as “~~ Definitely high risk of bias (red)”. When a positive control showed an effect or was not included, but the treatment altered TH or TSH, the study was marked as “++ Definitely low risk of bias (dark green)” or “+ Probably low risk of bias (light green)”, respectively. If a positive control was not included and the treatments did not show effects, then, the study was classified as “~ Probably high risk of bias (orange)”.
6. Timing of sampling after ending the treatment for TH/TSH measurements and duration of the exposure. When significant changes of TH/TSH levels (including T3) were observed and the timing of sampling was in accordance with Organisation for Economic Co-operation and Development/Environment Protection Agency (OECD/EPA) test guidelines (specified in the methodology), we considered a study as “++ Definitely low risk of bias (dark green)”. If there were ambiguities in relation to compliance with test guideline recommendations (no specification of guidelines in the methodology), but significant effects were observed, we rated a study as “+ Probably low risk of bias (light green)”. Studies were marked as “~ Probably high risk of bias (orange)” when no significant changes were observed and there were ambiguities regarding adherence with test guidelines. “~~ Definitely high risk of bias (red)” studies with no changes and without adherence to guidelines (35).
7. Use of contemporaneous or historical controls as a comparator (negative control) of hormonal changes. When contemporaneous negative controls were used, we rated a study as “++ Definitely low risk of bias (dark green). When data from historical controls were used as a comparator, the study was evaluated as “~~ Definitely high risk of bias (red)”, as identical test conditions cannot be guaranteed. (See discussion in Vandenberg et al., 2020 (36)).”. The categories “+ Probably low risk of bias (light green)” and “~ Probably high risk of bias (orange)” were not used.
8. Use of soy-free animal feed. Soy-containing feeds can produce alterations in the hormonal levels, potentially mask the effects of EDCs (37) and can elicit goitrogenic effects in rats due to isoflavones (38, 39). When the diet contained soy-derived components or alfalfa, the study was rated as “~~ Definitely high risk of bias (red)”. In cases where we were unable to trace the components of the diets, we scored the study as “+ Probably low risk of bias (light green)”. When the authors specified the diet as “soy-free”, then the study was ranked “++ Definitely low risk of bias (dark green)”.

For the overall study evaluation, we adopted the decision rules detailed in EFSA (40) which encompass 3 Tiers. We placed studies in the highest confidence level (Tier 1, green) when all 8 key elements were scored ++ (definitely low risk of bias) OR + (probably low risk of bias) AND no more than 1 question not addressing key elements was scored ~ (probably high risk of bias) or ~~ (definitely high risk of bias). A medium confidence level (Tier 2, orange) was used for all combinations not covered in Tier 1 or Tier 3. The lowest confidence level (Tier 3, red) was reserved for studies where any one of the 8 key elements was rated ~ or ~~ OR more than 50% of the questions not addressing these key elements were scored ~ or ~~.

The RoB scheme was piloted with a random sample of 10 studies which were scored independently by 3 different team members. Any conflict regarding RoB outcomes was reviewed and resolved after discussion between the team members.

### 2.4 Data synthesis

Due to data heterogeneity, it was not possible to conduct a quantitative meta-analysis. Instead, the findings of selected studies were summarized in a narrative synthesis. For the data synthesis we compiled a list of test compounds associated with a specific serum TH/TSH pattern as well as a list of chemicals repeatedly assessed in different studies to evaluate the consistency of TH/TSH effects. All other outcomes were summarized (e.g., enzymatic activities, gene expressions) to examine potential MoAs. We have included studies rated in Tier 1, 2 and 3 in order to evaluate all available data, whilst highlighting selection and performance factors which could influence the assessment.

## 3 Results

### 3.1 Study selection

Outcomes at the different stages of our literature search and screening process are shown in the PRISMA flow chart (**Figure 1**) (41). Initially, a total of 30,282 records were retrieved from three different databases (Pubmed, Web of Science, Scopus). To this initial search, we included the update in January 2024 with an additional 3,959 records. Duplicates were removed leaving 25,107 records which were manually screened for relevance (titles and abstracts). Of these, 23,870 records were excluded as they did not meet our eligibility criteria or because they included one or more of the following: i) exposure factors other than chemicals, ii) incomplete T4 and TSH data; iii) studies in systems other than *in vivo* experiments, iv) epidemiological studies; v) records that were not primary, peer reviewed research papers.

**Figure 1:**
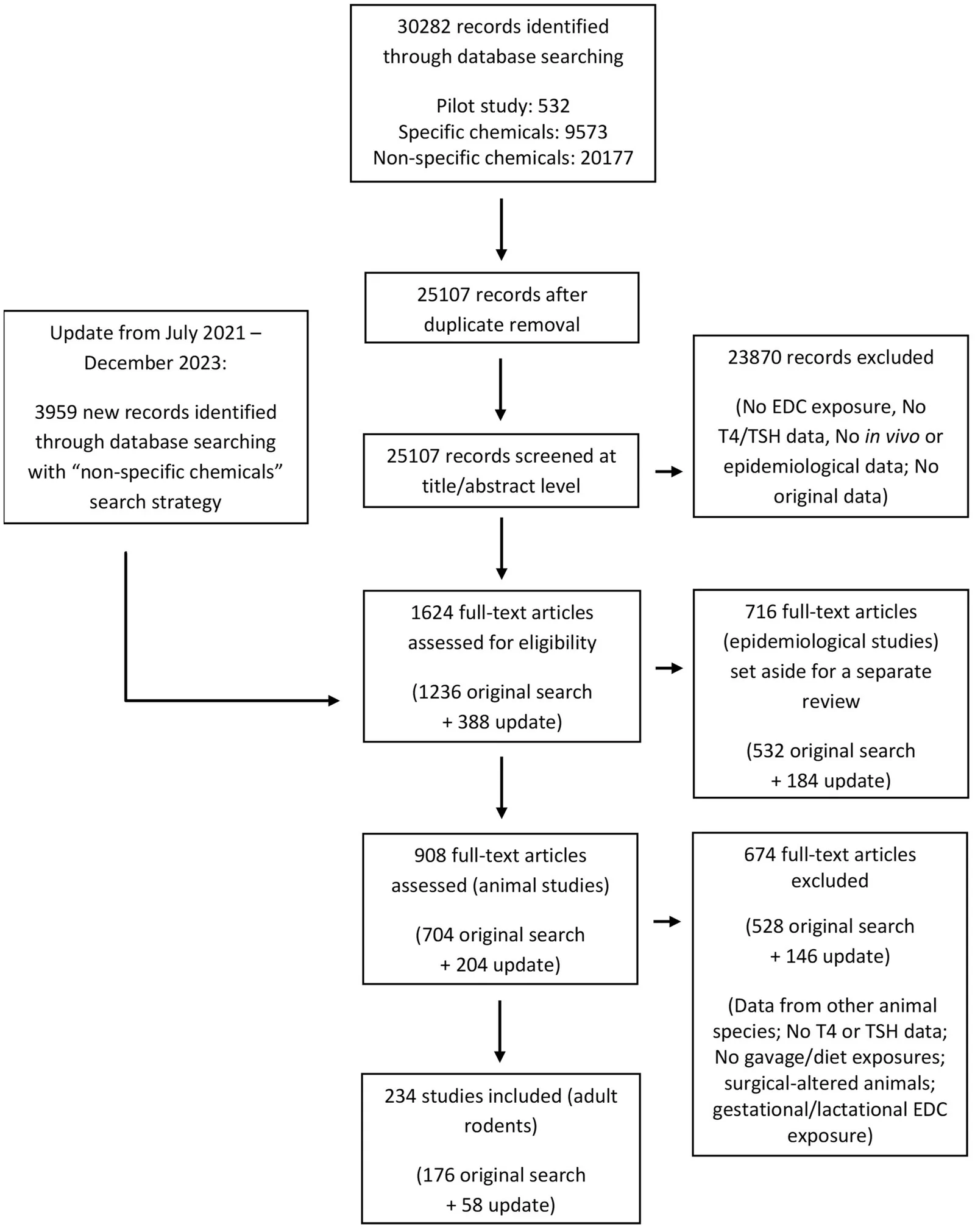
PRISMA flow diagram. Depiction of the different stages of our literature search.

The remaining 1,236 articles (+388 from update) were screened in full-text in accordance with our PECO criteria (**Table 1**). At this stage, we decided to set aside 532 (+184 from update) full-text records for a follow-up evidence mapping focusing on human epidemiology. Of the remaining 704 studies (+204 from update), 528 full-text articles were excluded (+146 from update) for the following reasons: data from non-rodent animal species; no T4 and/or TSH data; no oral exposures; surgically altered animals, gestational and/or lactational chemical exposure (**Supplementary Material 3**). Finally, the remaining 234 articles (176 plus 58 articles from update) of adult rodent studies were included in the data extraction and evaluation (see **Supplementary Material 4** for metadata, 5 for data from multiple-dose studies and 6 for single-dose studies). From the 234 articles, 139 were multidose experiments whereas 106 articles reported only a single dose. Eleven articles reported multidose and single dose experiments in the same study and were analyzed in duplicate.

### 3.2 Risk of bias evaluation

We conducted a RoB analysis to evaluate the internal validity of eligible studies. To assess the RoB for each study we extracted and tabulated characteristics that could be associated with potential bias. For studies which reported the effects of multiple chemicals, RoB was assessed for each tested compound; therefore, the unit of evidence was the chemical and not the study. Thus, for the 139 studies which conducted multi-dose experiments, we have 184 units of evidence (**Supplementary Material 7**).

The number of units of evidence in each category for each question of the bias assessment are shown in **Table 3**. For key elements, the number of animals per dose group had the lowest risk of bias with 155 units of evidence as did the use of contemporaneous controls with 184 units of evidence. High risk of bias was observed for the inclusion of phytoestrogens in the diet with 83 units of evidence ranked with “Definitely high risk of bias”. Regarding the T4/TSH quantification methods, we found several papers with inadequate descriptions of method details and validation, or the use of methods/kits designed for human samples without rodent adaptation. A total of 132 units of evidence were scored with “probably high risk of bias” for T4 analytical methods and 89 for TSH.

**Table 3:** Ratings of each unit of evidence for all the questions involved in the RoB analysis for studies with two or more chemical doses.

RoB questions	++	+	~	~~
	Definitely low risk of bias	Probably low risk of bias	Probably high risk of bias	Definitely high risk of bias
Key elements				
Were reliable and sensitive methods used for the T4 quantification?	1	51	132	0
Were reliable and sensitive methods used for the TSH quantification?	8	87	89	0
Was exposure sufficiently characterised?	54	127	3	0
Was the number of animals per dose group sufficient?	155	10	19	0
Was a positive control included?	12	150	15	7
Were measurements collected at a suitable timepoint when we compare control and treated groups? Was the duration of the treatment adequate?	29	134	21	0
Have the authors evaluated the hormonal changes in relation to contemporaneous controls or historical controls?	184	–	–	0
Was the diet free of phytoestrogens or/and goitrogens?	12	89	–	83
Other elements				
Were animals randomly allocated to dose groups?	100	84	0	0
Was allocation to dose groups adequately concealed?	0	184	0	0
Were all experimental animals of similar age, strain, health status and source?	105	70	9	0
Were experimental conditions identical across study groups?	42	142	0	0
Were research personnel blinded to study groups?	0	184	0	0
Were outcome data complete without attrition or exclusion?	74	110	0	0
Were the factors that might influence the variability of TH measurements considered?	39	142	3	0
Have all study outcomes been reported?	164	14	4	2
Have funding sources and conflicts of interest been reported?	80	–	–	104
Were statistical methods reported in the study appropriate and consistent?	16	158	10	0

Ratings of each unit of evidence for all the questions involved in the RoB for studies with two or more chemical doses. Shadings are related to the number of items; higher numbers of studies are shaded with darker colours. Symbols: ++ Definitely low risk of bias, + Probably low risk of bias, ~ Probably high risk of bias, ~~ Definitely high risk of bias.

Of the 184 units of evidence evaluated from multi-dose studies, 24 were rated as Tier 1 (high-confidence study) as all key questions were scored as “definitely low risk of bias” or “probably low risk of bias” as well as most of the remaining questions. Only one record was assigned in Tier 2 (Cadima #22505) owing to the absence of reported funding sources and/or conflicts of interest and insufficient description of the statistical methods. Finally, 159 units of evidence were classed as Tier 3 as one or more of the key elements scored “definitely high risk of bias” or “probably high risk of bias”. Most of the units of evidence did not meet our quality criteria for T4/TSH quantification and use of soy-containing diets. Only 19 studies included less than 5 animals per experimental group and 21 articles did not provide sufficient evidence on treatment duration and whether measurements were made at a suitable time point (**Supplementary Material 8**). Another shortcoming outside the key questions was the absence in reporting of funding sources and/or conflicts of interest in 104 units of evidence. A similar analysis was performed for the articles with single-dose treatments with a total of 106 articles (167 units of evidence) evaluated (**Supplementary Materials 9, 10**).

### 3.3 Characteristics of eligible studies and T4 - TSH response patterns

Most studies were conducted in rats; however, 17 out of 234 studies used mice (Cadima ##18130, 308, 2391, 1897, 16851, 1929, 22911, 22640, 1081, 744, 18670, 16386, +update: ##113, 216, 354, 876, 4901). Test chemicals were all administered orally, by gavage, diet or via drinking water at life stages ranging from post lactation (from PND21) to full adult. Only 3 studies started dosing via diet before PND21 (study #281 with BPA at PND15, #1458 with PCB153 at PND3, study #5400 from the update with DEHP with 2-week-old rats). Only one study (#17214) used rats at PND21. We also included the studies #509, in which pups received DDT via maternal milk during the lactation period and orally after weaning, before being sacrificed at 6 weeks-old; and #4901 in which dosing was dermal but not subcutaneous or intraperitoneal.

Regarding hormone measurements, the studies mainly measured serum T4, T3 and TSH. Both T4 and TSH were required to meet our eligibility criteria. We included T3, fT3 and fT4 where available. We also included 21 articles in which fT4/fT3 were measured instead of T4/T3 (Cadima ##254, 1008, 3844, 5901, 288, 104, 14492, 17214, 6761, 16312, 14492, 22260, 1833, 20133, 22616, 27670, 16718, +update: ##2276, 2707, 3726, 5782). In general, T4 and TSH were measured in serum as recommended by the American Thyroid Association (ATA) guidelines, to avoid interference with anticoagulants and ensure data reproducibility (2); however, 21 studies used plasma (Cadima ##307, 241, 2391, 1897,1008, 144, 1675, 1929, 1924, 2237, 22735, 20773, 385, 280, 978, 9232, 211, 1833, 26342, 13395, +update: ##208, 401, 534, 927, 1168, 5492). Percentage changes, calculated to enable comparability across studies, are available in **Supplementary Materials 5, 6** with further details for multi-dose and single-dose studies, respectively.

In addition, the search retrieved 12 articles using non-rodent animal models (mammalian species). Those studies used monkeys, rabbits, sheep, guinea pigs, polecats, cats, minipigs, calves and goats. The test compounds were the plastic additive ethylene-bis-(oxyethylene)-bis-(3-tert-butyl-4-hydroxy-5-methylhydrocinnammate), PFOS, cadmium, mercury, vitamin C, 3,4,3’,4’-tetrachlorobiphenyl, selenium, fluoxetine hydrochloride, phytosterols, povidone-iodine, lysozyme and two dietary supplements: olive and flax seed oils. Because of the difference between models and the difficulty of comparing such results with rodents, we excluded them from the present evidence map (data are available in **Supplementary Material 14**).

### 3.4 Patterns of altered T4 - TSH levels

Although we extracted data regarding all the TH (T3, T4, fT4, fT3), only the T4/TSH response patterns observed in the adult rodents were used to group the chemicals, as follows: decrease of serum T4 levels in combination with an increase in TSH (summarized results in **Table 4**); decrease of serum T4 with no changes in TSH (**Table 5**), no change in either T4 or TSH (**Table 6**) and mixed patterns, with either unchanged or increased T4 and varying TSH changes (**Supplementary Material 12**). In addition, we grouped multiple studies which assessed the same chemical to evaluate the consistency of response patterns among studies (**Table 7**). **Tables 4**-**7** report the chemicals together with their CADIMA reference number, life stage at dosing, the duration of treatment as well as exposure concentrations. Additional information, such as the life stage at time of sampling can be accessed in **Supplementary Material 4**. We also included studies scored as Tier 3 due to the restrictive RoB rating criteria resulting in a low number of studies scored as Tier 1 or 2. This would have resulted in the omission of a substantial set of test chemicals.

**Table 4:** Summary of T4 – TSH response patterns with chemicals producing T4 decrements and TSH increases.

↓T4 ↑TSH				
Chemical name	CADIMA reference number	Life stage at start of dosing	Treatment duration	Exposure concentration (mg/kg bw/d)
Arsenic	3067	Adult	36 w	2.5 - 10
Bismerthiazol	242	3.1 w	20 d	10 - 40
BPA	281	2 w	15 d	0.02, 0.04
Brom. vegetable oil	1727	6 w	90 d	0.002 - 0.5%
Cyproterone acetate	14746	10 w	15 d	1 - 100
DE-99*	5901	4 w	15 d	30 - 120
DE-209	494	7 w	28 d	5 - 500
Di-n-butyl phthalate (DBP)	5003	6 w	2 w	50 - 500
Doxylamine succinate	22640	Adult	7, 15 d	750, 1500
Glyphosate-based*	1008	Adult	60 d	126, 315
Hexachlorobenzene	1675	Adult	28 d	4, 16
Hexachloronaphthalate*	6761	Adult	2, 4, 13 w	0.03 - 0.3
Ketoconazole	215	7 w	28 d	6.25 - 100
Kojic acid	22026	6 w	4 w	0.01 - 2%
Melamine cyanuric acid	18670	4 w	4 w	10 - 40
Nonylphenol*	288	5–6 w	28 d	30 - 270
	104	4 w	15 d	40 - 200
Polybrominated biphenyls (PBB)	2237	Adult	20 d	3, 6
PCB126	2199	Adult	1 d	0.0075 - 0.275
Perchlorate	105	3.3 w	30 d	62.5 - 500
Phenobarbital	17141	7 w	14 d	5 - 80
	1081	7 w	21 d	600–2400 ppm/d
Pongamia Oil	2442	6–7 w	90 d	2.5 - 10%
PTU	385	6.2 - 6.6 w	28 d	0.1 - 10
Sodium chlorate	745	Adult	4, 21, 90 d	0.125–2 g/L
Spironolactone	18084	5 w	2, 4, 13 w	6 - 500
3,3',4,4'-tetrachloroazobenzene (TCAB)	1174	6 w	21 d, 13 w	0.1 - 30
2,3,7,8-Tetrachlorodibenzo-p-dioxin (TCDD)	9232	Adult	1 d	0.00625 - 0.1
	541	6 w	1 d	0.001 - 0.004
Testosterone	27249	3 w	20 d	0.05 - 1
Thiazole-Zn	35	7 w	6 d	8 - 40
Thiazopyr	8918	11–23 w	56 d	10 - 3000
Thiodiazole copper	243	3.1 w	20 d	4 - 30
Tributyltin	16851	3 w	45 d	0.0005 - 0.05
Triclosan	744	12 w	50 d	1 - 100
	4901	4–6 w	20 w	0.3, 1.5% w/v
Vinclozolin	1710	7 w	28 d	3.125 -200
3,3’,4,4’-tetrachloroazoxybenzene	1110	6 w	13 w	0.1 - 10
3-amino-1,2,4-triazole	504	8 w	28 d	5 - 125
4-4’-butylidenebis (2-tert-butyl-5-methylphenol)	214	8 w	28 d	5 - 125

Shown are records for test compounds with corresponding Cadima reference numbers. Most of the studies reported doses of test chemicals as mg/kg bw/day, except for studies in which chemicals were administered via drinking water with the doses expressed as parts per million (ppm) or percentage (%). Asterisk (*) indicates studies where FT4 was measured instead of T4. Studies ranked as Tier 1 are highlighted in green. Studies with mice instead of rats were shadowed in grey. Life stage was indicated as “Adult” where no specific age was provided. w, week(s); d, day(s). See **Supplementary Material 11** for details. Symbols: ↑ (increase); ↓ (decrease).

**Table 5:** Summary of T4 – TSH response patterns with chemicals producing T4 decrements with no TSH changes.

↓T4 ↔TSH				
Chemical name	CADIMA reference number	Life stage at start of dosing	Treatment duration	Exposure concentration (mg/kg bw/d)
Aroclor 1242	1029	Adult	7 d	4 - 32
Aroclor 1254	1029	Adult	7 d	4 - 32
	1081	7 w	21 d	10–300 ppm/d
Aroclor 1260	808	8–9 w	10 d	0.025 - 156
BPA	13283	7 w	42 d	20, 100
Bromkal 70-5	1929	7 w	14 d	18, 36
DBP	14746	10 w	15 d	250 - 1000
Di(2-ethylhexyl) phthalate (DEHP)	13286	8 w	2 w	50, 500
	276	3,6 w	30 d	250 - 750
	13283	7 w	42 d	150, 750
	112	7 w	28 d	5 - 500
DE-47	1525	9 w	8 w	0.001 - 1
	1153	1,4 w	1 d	0.001 - 0.01
DE-71	33	7 w	4 d	0.3 - 300
	1	3.1-3.3 w	5, 20/31 d	3 - 60
DE-79	33	7 w	4 d	0.3 - 100
Equol	1291	8 w	5 d	100, 250
Flutamide	14746	Adult	15 d	5 - 100
Fluopyram	1897	10 w	3 d	100, 300
Hexachlorobenzene	144	Adult	4 w	1 - 500
Isobutylparaben	2015	3 w	20 d	62.5 - 1000
Isopropylparaben	2015	3 w	20 d	62.5 - 1000
Linuron	14746	Adult	15 d	25 - 150
Methylparaben	2015	3 w	20 d	62.5 - 1000
Myclobutanil	165	7 w	4, 30, 90 d	100 - 2000
Nicofluprole	401	6–7 w	90 d	30 - 2000
Nonylphenol*	5782	Adult	17, 34 w	0.4 - 40
Oxyfluorfen	1976	9 w	7 d	0.8125 - 62.5
PCB95	1029	Adult	7 d	2 - 16
PCB99	1029	Adult	7 d	2 - 16
PCB118	1029	Adult	7 d	2 - 16
	1457	8 w	14, 31, 53 w	0.01 - 4.6
PCB126	1029	Adult	7 d	0.0025 - 0.04
	1457	8 w	14, 31, 53 w	0.00001 - 0.001
PCB153	1458	0.4 w	4 d	0.025, 2.5
	1457	8 w	14, 31, 53 w	0.01 - 3
2,3,4,7,8-pentachlorodibenzofuran(PeCDF)	1457	8 w	14, 31, 53 w	0.000006 - 0.0002
PFOS	700	Adult	91 d	1.7–15 mg/L
1,1-Dichloro-2,2-bis(p-chlorophenyl)ethylene (p,p'-DDE)	14746	Adult	15 d	50 - 300
Propylparaben	2015	3 w	20 d	62.5 - 1000
PTU	386	3.3 w	30 d	0.01, 1
Sulfamethazine	24503	3–9 w	12, 18, 24 m	10 - 2400
Tartrazine	208	6–7 m	15, 30 d	9.6, 96
Tetrabromobisphenol A (TBBPA)	993	8 w	30, 90 d	100 - 1000
TCDD	1029	Adult	7 d	0.00014 - 0.0039
	2174	Adult (?)	1, 2, 4, 8, 16, 28/32 d	0.025, 0.125
	1457	8 w	14, 31, 53 w	0.000003 – 0.0001
Triclosan	275	Adult	8 m	2.35 - 37.5
	280	3.3 w	31 d	50 - 200
	1098	3.1 w	20 d	9.375 - 150
	1336	3.9 – 4.1 w	4 d	30 - 1000
	2030	3,3 w	31 d	3 - 300
Rose Bengal	16386	8 w	2 w	0.13, 0.25%
Vinclozolin	3257	8 w	10 w	40 - 1000
	14746	10 w	15 d	10 - 300
*Vitellaria paradoxa*	18132	10–14 w	30 d	50 - 400
Xylazine hydrochloride	15208	5 w	26 w	250 - 1000
6:2 chlorinated polyfluorinated ether sulfonate (F-35B)	907	9 w	28 d	5 - 100

Symbols as in **Table 4**. Details in **Supplementary Material 11**. Symbols: ↔ (no change), ↓ (decrease).

**Table 6:** Summary of studies where T4 - TSH changes were not observed.

↔T4 ↔TSH				
Chemical name	CADIMA reference number	Life stage at start of dosing	Treatment duration	Exposure concentration (mg/kg bw/d)
Acrylamide	13789	3.3 w	37 d	2.5, 5
Atrazine	1232	3.3 w	53/54 d	12.5 - 200
	4311	3.1 w	19 d	12.5 - 200
Benzoic acid	5654	10 w	10–11 w	7500 - 15000
Boron	2736	Adult	3 w	10, 20
BPA	113	6–8 w	5 w	0.002 - 20
Butylparaben	2015	3 w	20 d	62.5 - 1000
Carbendazim	1533	Adult	15 w	150 - 600
Cadmium	6666	3 w	20 d	1–100 mg/L
Chlorpyrifos	6838	3 m	5 d	0.01 - 10
Curcumin	208	6–7 m	15, 30 d	3.85, 38.5
DBP	880	4 w	30 d	100, 500
DE-47	1924	7 w	14 d	1 - 18
DE-83R	33	4 w	4 d	0.3 - 100
Deethylatrazine; Deisopropylatrazine; Diaminochlorotriazine	18277	3.3 w	30/31 d	12.5 - 200
Diethylstilbestrol	27249	3 w	20 d	0.0002, 0.001
Ethylparaben	2015	3 w	20 d	62.5 - 1000
Fulvic acid	26342	Adult	26 d	0.1 - 0.8%
Genistein	817	4.9 w	7 d	1, 5
Humic acid	26342	Adult	26 d	0.1 - 0.8%
Methyl tert-butyl ether	28070	7 w	28 d	250 - 1500
NaNO_3_	159	10 w	8 w	66 - 300
PCB28	2211	Adult	90 d	50–50000 ppb
Perchlorate	7512	7 w	7 d	0.1–10 mg/L
Saisentong	64	3.1 w	20 d	5 – 25
TBBPA	113	6–8 w	5 w	0.002 - 20
Soybean protein	18846	Adult ()?	12, 18, 24, 30 m	10, 40%
3-(dibutylamino)phenol	214	8 w	28	30 - 300

Symbols as in **Table 4**. Details in **Supplementary Material 12**. Symbols: ↔ (no change).

**Table 7:** Consistency of response patterns across studies. Overview of the test compounds repeatedly assessed in different studies.

Chemical name	CAD. ref.	Start of dosing	Treatment duration	Exposure concentration (mg/kg bw/d)	T4	TSH
Amiodarone	10593	Adult	4, 7 w	5, 15, 45	▲/↔	**▼**/↔
	17368	7–9 w	6 w	3	**▲**	**▲**
Aroclor 1254	1029	Adult	7 d	4, 8, 16, 32	**▼**	↔
	1081	7 w	21 d	10, 30, 100, 300 ppm/d	**▼**/↔	▲/↔
	1929	7 w	14 d	10	**▼**	↔
	321	Adult	7 d	100	**▼**	↔
	861	Adult	30 d	2 mg/ml	**▼**	**▲**
	2361	Adult	7 d	100	**▼**	↔
	1924	7 w	14 d	4	**▼**	↔
Arsenic	3067	Adult	36 w	2.5, 5, 10	**▼**	**▲**
	876	8 w	30 d	0.2, 2, 20	**▼**	**▼**
Atrazine	4311	3.1 w	19 d	12.5, 25, 50, 100, 200	↔	↔
	1232	3.3 w	30 d	12.5, 25, 50, 100, 150, 200	↔	↔
BPA	17871	3 m	30 d	50	**▼**	**▲**
	281	2 w	15 d	0.02, 0.04	**▼**	**▲**
	13283	4 w	42 d	20, 100	**▼**	↔
	113	6–8 w	5 w	0.002, 0.02, 2, 20	↔	↔
BPS	113	6–8 w	5 w	0.002, 0.02, 2, 20	↔	▲/↔
	18130	7–8 w	5 m	0.1	↔	↔
Cadmium	6666	3 w	20 d	1, 10, 100 mg/L	↔	↔
	1302	3 m	4 w	50 mg/L	**▼**	**▲**
	5977	Adult	6 w	3	**▼**	**▲**
Carbendazim	1533	Adult	15 w	150, 300, 600	↔	↔
	5633	Adult	8 w	100	**▼**	**▲**
Carbimazole	3887	3 m	2 m	6	**▼**	**▲**
	1209	Adult	3 w	83	↔	↔
	615	Adult	60 d	0.05	**▼**	**▲**
Chlorpyrifos	6120	Adult	14 d	5	↔	**▼**
	14344	Adult	11 w	10.6	↔	↔
	6838	3 m	5 d	0.01, 0.1, 1, 10	↔	↔
	279	Adult	30 d	6.75	**▼**	↔
	292	60 d	1 d	0.1	**▲**	▲/↔
	5492	Adult	6 w	6.7	**▼**	**▲**
Corn oil	21808	Adult	90 d	?	↔	↔
	6120	Adult	14 d	1 ml/200 g	↔	**▼**
	3726*	3 m	30 d	5 ml/kg bw/d	↔	↔
Curcumin	208	6–7 m	15, 30 d	3.85, 38.5	↔	↔
	16261	Adult	4 w	100	↔	↔
	3726*	3 m	30 d	15	↔	↔
Cyanide	971	7 w	2, 56, 90 d	3, 10, 30, 100, 300	**▼**/↔	↔
	22260*	Adult	56 d	2	**▲**	**▼**
DE-47	1525	9 w	8 w	0.001, 0.03, 1	**▼**/↔	↔
	1924	7 w	14 d	1, 6, 18	↔	↔
	1153	1.4 w	1 d	0.001, 0.005, 0.010	**▼**/↔	↔
	1929	7 w	14 d	18	**▼**	↔
DE-99	3844	4 w	10, 15, 20, 30, 40 d	60	**▼**/↔	▲/↔
	5901*	4 w	15 d	30, 60, 120	**▼**/↔	**▲**
DE-71	33	4 w	4 d	0.3, 3, 10, 30, 100, 300	**▼**/↔	↔
	1	3.1-3.2 w	5, 20/31 d	3, 30, 60	**▼**/↔	**▲**/↔
DBDPE	22911	Adult ()?	30 d	5, 20, 100, 200	↔	**▲**/↔
	494	7 w	28 d	5, 50 500	↔	**▲**/↔
DBP	14746	10 w	15 d	250, 500, 750, 1000	**▼**/↔	↔
	880	4 w	30 d	100, 500	↔	↔
	5003	6 w	2 w	50, 250, 500	**▼**	**▲**
DDT	23	Adult	6, 10 w	0.00189, 0.00777	▼/▲/↔	▼/▲/↔
	493	Adult	6, 10 w	0.002	**▲**/↔	**▼**/**▲**
	509	0,1 w	6 w	0.00371	**▼**	**▲**
DEHP	6312	5–7 w	3, 6 m	150, 300, 600	▼/▲/↔	**▼**/**▲**
	13286	8 w	2 w	50, 500	**▼**/↔	↔
	276	3.6 w	30 d	250, 500, 750	**▼**/↔	↔
	13283	4 w	42 d	150, 750	**▼**/↔	↔
	112	4 w	28 d	5, 50, 500	**▼**/↔	↔
	5400	2 w	90 d	150, 300, 600	**▼**/↔	**▼**/↔
	3726*	3 m	30 d	500	**▼**	↔
Deltamethrin	254*	7–8 w	1, 30 d	15	↔	↔
	308	33 w	28 d	3, 6, 12	**▼**/↔	**▼**/↔
	1829	Adult	28 d	2.56	**▼**	**▼**
Ethanol (EtOH)	1536	Adult	8 w	3 ml/d	↔	**▲**
	15949	Adult	3 w	20% calories	↔	↔
	19865	Adult	21 d	36% calories	**▼**	↔
	21096	Adult	3 w	5% (w/v)	↔	↔
	23848	Adult	40 d	10% (w/v)	**▼**	**▼**
	25420	Adult	4, 8 w	10% (w/v)	**▼**	**▼**
Ethinyl estradiol	1469	3,3 w	30 d	0.0007, 0.007	**▲**	**▲**/↔
	2015	3 w	20 d	1	↔	↔
	275	Adult	8 m	0.001	↔	↔
Estradiol	5782*	Adult	34 w	0.03	**▼**/↔	↔
	91	6 w	28 d	10 ppm	▲/↔	↔
Fipronil	21808	Adult	90 d	4.85	**▼**	↔
	307	Adult	14 d	3	**▼**	↔
	241	9 w	14, 28 d	3	**▼**	**▲**
	2391	4.4 w	30 d	95	↔	↔
Flutamide	14746	Adult	15 d	5, 20, 50, 100	**▼**/↔	↔
	27249	3 w	20 d	1, 5, 25	↔	▲/↔
	1291	8 w	5 d	100	**▼**	↔
Glyphosate	1008*	Adult	60 d	126, 315	**▼**	↔
	556	3,3 w	37, 67 d	0.5, 5	▲/↔	↔
Hexachlorobenzene	144	Adult	4 w	1, 10, 100, 500	**▼**/↔	↔
	1675	Adult	28 d	4, 16	**▼**	**▲**
	11495	Adult	1, 4 w	100 mg/100 g bw/d	↔	▲/↔
Iodine	1955	4 w	4, 8, 12, 24 w	3, 6, 10, 50x	↔	**▲**/↔
	27670	3 w	6 w	<0.05	**▼**	**▲**
Lead	651	Adult	4 m	233.25	↔	**▲**
	16312*	Adult	14 w	30 mg/kg bw/2 d	**▼**	**▲**
	314	6 w	28 d	0.1, 0.5, 1, 3, 7, 15	▲/↔	▲/↔
	5459	2–3 m	14 d	10, 25	▲/↔	↔
	2707*	Adult	8 w	10	**▼**	**▲**
Lithium	2044	Adult	3 w	0.3 gr/100 gr food	↔	**▲**
	21344	Adult	8 w	1100	**▼**	**▲**
Methylparaben	2015	3 w	20 d	62.5, 250, 1000	**▼**/↔	↔
	2044	Adult	3 w	0.4 gr/100 gr food	↔	↔
NaF	3024	Adult	35 d	40	**▲**	**▲**
	3090	4 m	6 w	20	**▼**	↔
Nonylphenol	288*	5–6 w	28 d	30, 90, 270	**▼**/↔	**▲**
	4293	8 w	14 w	2, 10, 50	**▼**/↔	▲/↔
	104*	4 w	15 d	40, 80, 200	**▼**	**▲**
	5782*	Adult	34 w	0.4, 4, 40	**▼**/↔	↔
PCB118	1029	Adult	7 d	2, 4, 8, 16	**▼**	↔
	1457	8 w	14, 31, 53 w	0.01, 0.03, 0.1, 0.22, 0.46, 1, 4.6	**▼**/↔	▲/↔
PCB126	1457	8 w	14, 31, 53 w	10, 30, 100, 175, 300, 550, 1000 ng/kg bw/d	**▼**/↔	▲/↔
	1029	Adult	7 d	0.0025, 0.005, 0.01, 0.02, 0.04	**▼**/↔	↔
	2199	Adult	5, 9, 22 d	0.0075, 0.075, 0.275	**▼**/↔	▲/↔
PCB153	1458	0.4 w	4 d	0.025, 2.50	**▼**	↔
	1153	1.4 w	1 d	0.005	**▼**	↔
	1457	8 w	14, 31, 53 w	0.01, 0.1, 0.3, 1, 3	**▼**	↔
Perchlorate	7512	7 w	7 d	0.1, 1, 10 mg/L	↔	↔
	208	8 w	60 d	35	**▼**	**▲**
	105	3.3 w	30 d	62.5, 125, 250, 500	**▼**/↔	▲/↔
PFOS	700	Adult	91 d	1.7, 5, 15 mg/L	**▼**	↔
	688	8–10 w	1 d	3, 15	**▼**	**▼**/↔
	1765	Adult	3, 7, 14, 20 d	3, 5	**▼**	▼/▲/↔
	4155	8 w	7 d	3	**▼**	↔
Phenobarbital	1503	11 w	2 w	75	**▼**	**▲**
	22640	Adult	7, 15 d	375	**▼**	▲/↔
	1056	7 w	14 d	0.1 w/v	**▼**	↔
	17141	7 w	14 d	5, 20, 80	**▼**/↔	▲/↔
	1081	7 w	21 d	600, 1200, 1800, 2400 ppm/d	**▼**	▲/↔
	20246	Adult	3, 7, 14, 21, 30, 45, 60, 90 d	1200	**▼**/↔	↔
	22505	Adult	7, 28 d	75	↔	▲/↔
	91	6 w	28 d	500	↔	↔
Pregnenolone	1081	7 w	21 d	250, 500, 1000, 2000 ppm/d	▲/↔	**▲**
	321	Adult	7 d	1000	**▼**	**▲**
	2361	Adult	7 d	800	**▼**	**▲**
	20246	Adult	3, 7, 14, 21, 30, 45, 60, 90 d	500	**▼**/↔	▲/↔
PTU	386	3.3 w	30 d	0.01, 1	**▼**/↔	↔
	385	6.3-6.6 w	28 d	0.1, 1, 10	**▼**/↔	**▲**
	861	Adult	30 d	10 mg/ml	**▼**	**▲**
	20246	Adult	3,7, 14, 21, 30, 45, 60, 90 d	30	**▼**	▲/↔
	688	8–10 w	7 d	10	**▼**	**▲**
	2291	7 w	30 d	0.05%	**▼**	**▲**
	2581	Adult	4 w	10	**▼**	**▲**
	2704		45 + 30 d	15	**▼**	**▲**
	2731	10–12 w	30 d	0.05%	**▼**	**▲**
	2736	Adult	3 w	0.05%	**▼**	**▲**
	3119	7 w	4 w	1mg/100 g	**▼**	**▲**
	4815	6 w	30 d	6	**▼**	**▲**
	216	3–7 w	4 w	100 ug/L	**▼**	**▲**
	91	6 w	28 d	50	**▼**	**▲**
Tamoxifen	17214	3 w	7 w	5	**▲**	↔
	27249	3 w	20 d	0.01, 0.05, 0.2	↔	▲/↔
TBBPA	6666	3 w	20 d	50	↔	↔
	993	8 w	13 w	100, 300, 1000	**▼**/↔	↔
	1019	12 w	5 d	250	**▼**	↔
	113	6–8 w	5 w	0.002, 0.02, 2, 20	↔	↔
TCDD	1457	8 w	14, 31, 53 w	3, 10, 22, 46, 100 ng/kg bw/d	**▼**/↔	▲/↔
	1029	Adult	7 d	0.00014, 0.00043, 0.0013, 0.0039	**▼**/↔	↔
	2174	?	1, 2, 4, 8, 16, 28/32 d	0.025, 0.125	**▼**/↔	↔
	9232	Adult	1 d	0.00625, 0.0125, 0.025, 0.05, 0.1	**▼**	**▲**
	541	6 w	1 d	0.001, 0.002, 0.004	**▼**/↔	▲/↔
	2412	Adult	1 d	0.025	**▼**	**▲**
Thiazole-Zn	1769	3.1 w	20 d	40, 100, 200	**▲**	▲/↔
	35	7 w	6 d	8, 16, 40	**▼**/↔	▲/↔
Tributyltin	29984	Adult	30 d	5	**▼**	**▲**
	102	Adult	40 d	200, 1000 ng/kg bw/d	**▼**/↔	▲/↔
	16851	3 w	45 d	0.0005, 0.005, 0.05	**▼**/↔	**▲**
	107	4 w	15 d	100 ng/kg bw/d	↔	**▼**/↔
Triclosan	275	Adult	8 m	2.35, 4.69, 9.375, 37.5	**▼**/↔	↔
	744	12 w	50 d	1, 10, 100	**▼**/↔	▲/↔
	280	3.3 w	21 d	50, 100, 200	**▼**/↔	↔
	1098	3.1 w	20 d	9.375, 37.5, 75, 150	**▼**/↔	↔
	1336	3.9-4.1 w	4 d	30, 100, 300, 1000	**▼**/↔	↔
	2030	3.3 w	31 d	3, 30, 100, 200, 300	**▼**/↔	↔
	4901	4–6 w	20 w	0.3, 1.5% w/v	**▼**	**▲**
Selenium	27670	3 w	6 w	<0.05	↔	↔
	3726*	3 m	30 d	2	↔	↔
Sodium chlorate	7512	7 w	7 d	10, 100, 1000 mg/L	↔	▲/↔
	745	Adult	4, 21, 90 d	0.125, 1, 2 g/L	**▼**/↔	▲/↔
Soya oil	14344	Adult	11 w	2 ml/kg bw	↔	↔
	682	Adult	4 m	1	↔	↔
Soybean protein	18846	0 w	12, 18, 24, 30 m	10, 40%	↔	↔
	16718*	1 m	90 d	1.12, 2.64 g/100 ml	**▲**/↔	**▲**/↔
Vinclozolin	3257	6 w	10 w	40, 200, 1000	**▼**/↔	↔
	1710	7 w	28 d	3.125, 12.5, 50, 200	**▼**/↔	**▲**/↔
	14746	10 w	15 d	10, 75, 150, 300	**▼**	↔
Zinc	27670	3 w	6 w	3.5	↔	↔
	13395	3 w	42 d	< 1	**▼**	↔
	21344	Adult	8 w	227 mg/L	↔	↔
	1536	Adult	8 w	227 mg/L/d	↔	↔
ZnO-NP	5199	16–18 w	30 d	200	**▼**	**▼**
	2707*	Adult	8 w	85	**▼**	**▲**
3-methylcholanthrene (3-MC)	2361	Adult	7 d	200	**▼**/↔	↔
	321	Adult	7 d	250	**▼**	↔
	1081	7 w	21 d	62.5,125, 250, 500 ppm/d	**▲**/↔	**▲**

Overview of the test compounds repeatedly assessed in different studies (single dose studies are included as they can support multiple-dose studies). Cadima reference number with asterisk (*) means that fT4 was measured instead of T4. Units of evidence ranked as Tier 1 are highlighted in green. Mice studies are shadowed in grey. Life stage was indicated as “Adult” where no specific age was provided. m, month(s); w, week(s); d, day(s). For details regarding dosing, see **Supplementary Materials 5, 6**. Symbols: ▲ (increase);▼(decrease); ↔ (no change).

Changes in T4 or TSH levels were considered to be increased, decreased or no-change, based on whether differences were reported as statistically significant in the studies. Detailed TH/TSH outcomes and percentage change for each dose compared to controls can be found in **Supplementary Materials 11, 12**. **Supplementary Material 13** lists similar patterns for studies with only one dose group.

#### 3.4.1 T4 serum decrements accompanied by TSH increases

Decreased serum T4 with increased TSH consistent with the HPT-axis negative feedback loop was expected to be the most frequently observed pattern, but was only the second most frequently observed response pattern, with 40 units of evidence out of 184 (summarized in **Table 4**). Chemicals producing the greatest T4 decrease, together with an increase in TSH include: PTU (28 day dosing, #385), the highest dose of 3-amino-1,2,4 triazole (#504) and kojic acid (28 day oral administration, #22026), nonylphenol (15 day dosing, #104) and single administrations of TCDD (#9232), and doxylamine succinate (#22640).

Among the chemicals that showed a significant decrease in T4, but a lesser increase in TSH were: TCAB (21 days and 13 weeks dosing, #1174), 3,3’-4,4’ tetrafluoroazoxybenzene (13 weeks, #1110), PCB126 (9 and 22 days, #2199) and polybrominated biphenyls (#2237).

While most studies were performed on adult rats, several administered the test compounds directly to younger rats, including BPA at PND15 (#281), tributyltin (#16841) and testosterone (#27249) at PND21, bismerthiazol or thiodiazole copper at PND22 (##242, 243) and perchlorate at PND23 (#105).

Also, some studies measured fT4 instead of T4, following administration of hexachloronaphthalate (#6761), nonylphenol (##288, 104), the glyphosate-based pesticide Kalach (#1008) and DE-99 (#5901).

Bismerthiazol (#242), cyproterone acetate (#14746), DE-209 (#494), ketoconazole (#215), kojic acid (#22026), melamine (#18670), spironolactone (#18084), thiazole-zinc (#35), thiazopyr (8918), the pesticide 3-amino-1,2,4-triazole (#504) and the antioxidant 4-4’-butylidenebis(2-tert-butyl-5-methylphenol) (#214) exhibited a dose-dependent decrease in T4. Testosterone significantly altered TSH levels at the mid-dose, whereas no significant effect was observed at the highest dose (#27249). A single oral administration of TCDD or PCB126 (#2199) were sufficient to induce TH/TSH alterations in rats (##9232, 541). However, some discrepancies were observed for different sampling times. For example, rats exposed to a single oral bolus of PCB126 showed TSH unaltered after 5 days, but TSH increased when the animals were sampled on day 9 and 22 (#2199). Similar responses were observed for different treatment durations with sodium chlorate (#745) or spironolactone (#18084).

#### 3.4.2 T4 serum decreases, but without accompanying TSH increases

The most frequently observed pattern was decreased serum T4 without corresponding increases in TSH **Table 5**, with a total of 58 units of evidence out of 184. As commented before, most of the studies were performed on adult rats, except the study with DE-47 which started direct exposure at PND10 (#1153) and PCB153 at PND3 (#1458). In addition, a few studies dosed rats around weaning, such as with parabens at PND21 (#2015), and with PTU (#386), triclosan (##280, 2030) and DE-71 (#1) at PND22/23.

Major decrements of T4 were shown with Aroclor 1254 (#1029), PCB99 and 118 (##1029, 1457), TCDD (#2174), DE-71 (#1), nicofluprole (#401), triclosan (#2030) and vinclozolin (#14746). We observed a dose-response T4 pattern for most test compounds with a few exceptions, such as for DE-47 (#1153), isopropylparaben, propylparaben (#2015) and nonylphenol (#5782) where only the medium dose significantly reduced T4. Although some compounds showed a considerable increase in TSH, these were not statistically significant, e.g. for specific doses of triclosan (#2030), Aroclor 1260 (#808), DE-71 (#1), PCB28 (#2211), PCB126 (#2199) and TCDD (##1457, 2174). The extract from *Vitellaria paradoxa* was the only compound that decreased TSH at the highest dose (#18132) (**Supplementary Material 11**). Again, different treatment durations produced dissimilarities, as in the case of myclobutanil (#165) or tartrazine (#208), where only the shortest dosage effectively reduced T4, or sulfamethazine, with significant effects after 18- and 24-month treatment but not after 12 months (#24503). PCB126 (#1457) showed changes in T4 with the shortest and longest exposures, as well as PCB153 (#1457). Of note, nonylphenol (#5782) reported significant fT4 decreases with the medium dose after 17-week treatment. After 34 weeks, only the highest dose reduced fT4.

#### 3.4.3 T4 - TSH changes were not observed and other patterns

Twenty-eight studies reported no significant changes in serum or plasma T4 and TSH following treatment with test chemicals but other relevant changes (**Table 6**). Despite the absence of changes in T4 and TSH, increases in T3 were observed in some cases, including chlorpyrifos (#6838) and carbendazim (#1533). In contrast, other compounds such as methyl tert-butyl ether (#28070), soybean protein (#18846) or 3-(dibutylamino) phenol (#214) decreased T3 levels (see **Supplementary Material 5** for T3 levels) with no change in T4 and TSH.

Less frequently reported patterns or chemicals with different TH/TSH responses within the same treatment (e.g. DDT (#23), DEHP (#6312), PFOS (#1765), triadimefon (#165)) are reported in **Supplementary Material 12**. Some examples of non-common TH responses include: i) increased T4 levels with decreased TSH with amiodarone (28-day treatment (#10593), *Ficus religiosa* (at 100 mg, #2731) or *Therminalia chebula* (#17834); ii) Decreased levels of both T4 and TSH after treatment with arsenic (#876), deltamethrin (6 mg, #308) or pentachlorophenol (#719); iii) no change in T4 but increased TSH, or increased T4 with no changes in TSH, observed with diethofencarb (#1653), diethyl phosphate (#11653), flutamide (#27249), *Quercus* extract (#1168) or 1,3-bis(2-pyrrolidone-1-yl)butane (28-day treatment #22505); iv) increased levels of T4 and TSH were reported with light crude oil (#211), famoxadone (#354), soybean protein (#16718); and finally, v) increased T4 with no change in TSH with lead (#5459) or thiazole-zinc (#1769).

### 3.5 Consistency of response patterns across studies

We observed some inconsistencies where the same compound showed diverse T4/TSH response patterns across different studies (**Table 7**). In many cases, these differences are likely due to differences in dosing, treatment duration and/or hormone measurement timepoints. However, some inconsistent patterns, such as with chlorpyrifos or BPA cannot be fully explained as they may involve other factors related to toxicokinetics or MoA. While two studies reported T4 decrements with increased TSH (##17871, 281) after BPA exposure, study number 113 did not detect any changes. In contrast, a fourth article (#13283) reported decreases in T4 without TSH changes. Six studies testing chlorpyrifos showed five different TH/TSH patterns, despite using similar doses. DBP is another example where despite similar dosing and treatment duration, two articles (##14746, 5003) both showed a reduction in T4 but unchanged or increased TSH. A similar pattern was observed with hexachlorobenzene, with both studies using similar exposure duration and dosing (##144, 1675). We observed that some test compounds exhibited higher variability for the TSH patterns while T4 appears more consistent. Whilst a higher variability in TSH is to be expected, also from a biological standpoint, it was more marked for some chemicals, such as amioradone, arsenic, DE-71, glyphosate PFOS, tributyltin or triclosan.

### 3.6 Relationship between response patterns and mechanism of action of thyroid hormone system perturbation

To investigate whether the TH/TSH response patterns can be related to a particular MoA, we compiled THS-related endpoint data where available. We also included outcomes from single-dose studies when the patterns concurred with the multi-dose studies (**Table 8**). Endpoints related to the mechanisms for THS disruption included histological analyses of the thyroid gland, THS -associated gene expression profiles (TH synthesis, regulation, transport and metabolism), enzymatic activities, TH-related protein levels or THR levels, among others. Studies which did not provide MoA information or showed different TH/TSH patterns (**Table 7**), were not included in **Table 8**. Detailed information for multi-dose and single-dose studies is provided in **Supplementary Materials 5, 6**.

**Table 8:** Relation of response patterns to mechanism of action (MoA) of thyroid hormone system perturbation.

↓T4, ↑TSH chemical (cad. ref. number)	Synthesis		Distributor proteins; TH transporters	Liver enzyme induction			DIOs	THr	TSHr TRHr	Thyroid histology	Other
	NIS	TPO		T4-UDPGT other UGTs	SULTs	Other markers					
Bismerthiazol (242)										Abnormal morphology	
BPA (*17871*, 281)										Abnormal morphology	
Brominated oil (1727)										Abnormal morphology	
Cadmium (*1302*, *5977*)										Abnormal morphology	
Carbimazole (*3887*, *615*)										Abnormal morphology	
Cyproterone (14746)										↔	
DE-99 (5901*, 3844*)		↑ TPO activity								Abnormal morphology	
DE-209 (494)	↓*Nis*	↓*Tpo*					↓*Dio1* *↑Dio2*		↓*Tshr* *↑Trhr*	Abnormal morphology	↑ TRH ↓TG production ↓*Tg* ↓*Pax8, ↓Ttf1* ↑*Ttf2*
Doxylamine succ. (22640)				↑ T4-UDPGT activity		↑ EROD, PROD activity					
Hexachloronaphthalate (6761*)						↑ CYP1A1 ↑CYP2B1 activity					
Ketoconazole (215)										↔	
Kojic acid (22026)										Abnormal morphology	↓ iodide uptake (thy)
Lead (16312*, 2707*)							*↓Dio1*			Abnormal morphology	
Melamine (18670)	*↓Nis*	*↓Tpo*	*↑Mct8 (hyp)* ↔*Mct8 (pit)*				↓*Dio2 (hyp, pit)*		*↓Tshr* *↓Trhr*	Abnormal morphology	*↓Tg*
Nonylphenol (288*, 4293, 104*)							↑ DIO 1,2,3 activity			Abnormal morphology	
PBB (**2237**)											↑Iodide uptake (thy)
Perchlorate (***208***, 105)	↑NIS *↑Nis*	↑TPO *↑Tpo*					*↓Dio1*		↑TSHr *↑Tshr* ↓TRH *↑Trh*	Abnormal morphology	↑TSHb ↑TSHa *↑Tsha* *↑Tshb* ↑TG *↑Tg*
Phenobarbital (*1503*, 22640, 17141, 1081)				↑T4 UGT activity ↑1A1 ↑1A6 ↑2B1		↑EROD ↑PROD				Abnormal morphology	
Pregnenolone *(321, 2361, 20246)*										Abnormal morphology	↑T4 biliary excretion ↔T4 clearance serum
PTU (385, *861,688, 20246*, 386, ***2291****, 2581, 2736, 3119, 4815, 216, 91*)	↑NIS									Abnormal morphology	↑ TRH ↓T4 thyroid ↑ TSH pituitary ↓ T4, ↔T3 liver, ↓ T4, T3 cerebellum
Spirolactone (18084)				↑T4 UGT activity						Abnormal morphology	↑ iodide uptake (thy)
TCDD (9232, 541, 1457, *2421*)				*↑ugt1*		*↑Cyp1a1*				Abnormal morphology	↑ iodide uptake (thy) ↑TSH (pit) ↑T4 (thy)
Thiazopyr (**8918**)				↑T4 UGT activity			↔DIO2			Abnormal morphology	↔ iodide uptake (thy) ↑T4 biliary excretion
Thiodiazole Cu (243)				↑						Abnormal morphology	
Tributyltin (***29984***, **16851**)	*Nis?*	↓TPO *↓Tpo*		↔ T4 UGT activity					*↑Tshr*	Abnormal morphology	↓TG *Tg?* ↓PAX8 *↓Pax8*
3-amino-1,2,4-triazole (504)										Abnormal morphology	

↓T4, ↔ TSH chemical (cad. ref. number)	Synthesis		Distributor proteins; TH transporters	Liver enzyme induction			DIOs	THr	TSHr TRHr	Thyroid histology	Other
	NIS	TPO		T4-UDPGT other UGTs	SULTs	Other markers					
Aroclor 1254 (1029, 1081, *1929*, *321, 2361*, *1924*)				↑ T4 UGT activity		↑EROD ↑PROD ↑MROD				Abnormal morphology	↑ T4 biliary excretion ↑ T4 clearance serum ↑ T4-glucuronide
Aroclor 1260 (808)						↑1A1, ↑1A2, ↑2B1, ↑2B2					
Aroclor 1242 (1029)						↑EROD ↔PROD					
Bromkal 70-5 (1929)				↑ T4 UGT activity		↑EROD ↑MROD PROD?					
DEHP (13286, 276, 13283, 112, 3726*)	*↑Nis* ↔NIS			*↑1a1* ↔1a6 ↑1A	*↑1a1* *↑2a1* *↔1a1* *↔1b1*			↔THrA ↔THrB	*↓Tshr* ↓TSHR *↑Trhr* ↑TRHR	Abnormal morphology	↑TRH (hyp)
DE-47 (1525, 1924, **1153**, *1929*)			↓ binding T4-TTR	↑ T4 UGT activity		↑EROD PROD? ↑MROD	*↑Dio1, 3* ↑DIO1, 3			↔	
DE-79 (33)				↑ T4 UGT activity		↑EROD ↑PROD					
Equol (1291)									*↓Tshb* *↓Trh*		
Fipronil (21808, ***307***, *2391*)	*↔Nis*	*↔Tpo*	*↔Slco1a1* *↔Slco4a1* *↔Slco1a5*	↑↑ T4 UGT activity *↑1a1*	*↑1b1* *↔1c3* *↓1a1*					Abnormal morphology	*↑Tg*
Flutamide (14746, *1291*)									*↓Tshb* *↔Trh*	↔	
Fluopyram (1897)				↑ T4 UGT activity		↑PROD			*↑Tshb*	Abnormal morphology	
Isopropyl, isobutyl, propyl2, methyl paraben (2015)										↔	
Linuron (14746)										↔	
Myclobutanil (165)				↑ T4 UGT activity		↑EROD ↑PROD ↔MROD				↔	
Nicofluopirole (401)				↑ T4 UGT activity							
PCB95 (1029)						↔EROD ↔PROD					
PCB99 (1029)						↔EROD ↑PROD					
PCB118 (1029, 1457)						↑EROD ↑PROD				Abnormal morphology	
PCB153 (**1458**, ***1153***, 1457)										Abnormal morphology	
PeCDF (1457)										Abnormal morphology	
PFOS (***4155***, **700**, *688*)	*↔Nis*	↔TPO		*↑1a1* *↔1a6*			*↓Dio1* (liver) *↑Dio1* (thy)		*↔Tshr*		↓T1Ac ↔T0 ↓ iodine-125 (serum, liver) ↑ iodine-125 (urine)
p,p’-DDE (14746)										↔	
Rose Bengal (16386)		↔TPO									
Sulfamethazine (24503)			↔TBG binding capacity								↔ T3 uptake (serum)
TBBPA (993, *1019*)			*↔Slc16a2* *↔Ttr*	*↑1a1* *↔2b1* *↔2b17*	*↓2a1* *↓1e1* *↔1a1*	*↑Cyp2b1, 2b2, 17a1, Cyp1b1, 3a1, 1a1, 19a1* *↔Car, ↔ Pxr* *↓Ppara, ↓Pparg*		*↑Thra* *↓Thrb*		↔	
Triclosan (275, 280, 1098, 1336, 2030)	↔NIS	↓TPO	*↔Oatp1a1* *↔Oatp1a4* ↔TTR	*↑/↔* T4 UGT activity ↑/↔1a1 *↔1a6, 2b5* *↑2b1*, ↑2B1	*↑/↔1c1* *↓/↔1b1* *↔1a1* *↑1e1* ↔2A1 ↑1E1	*↑Cyp1a1, 1a2, 2b1, 3a1* ↑CYP2B1 ↓EROD ↑PROD	↔DIO1,2 ↑DIO3	↔THrA1 ↑THrB1	↔TSHr ↑TRHr	Abnormal morphology	↔TRH plasma ↔TG
Vinclozolin (3257, 14746)										↔	
Xylazine hydrochloride (15208)										Abnormal morphology (mild)	
3-MC (*2361, 321*)											↑T4 biliary excretion ↔T4 clearance serum ↑ T4-glucuronide
F-35B (907)		↔TPO							↑TSHr	Abnormal morphology	

↔ T4, ↔TSH chemical (cad. ref. number)	Synthesis		Distributor proteins; TH transporters	Liver enzyme induction			DIOs	THr	TSHr TRHr	Thyroid histology	Other
	NIS	TPO		T4-UDPGT other UGTs	SULTs	Other markers					
Acrylamide (**13789**)	*↔Nis* *↔Pendrin*	*↑Tpo*	*↔Mct8 (Hyp, pit, liver)* *↑Mct8 (thy)*				*↔Dio1* *↔Dio2* *↑Dio3*	*↔Thra ↔Thrb (hyp, pit)* *↑Thra1* *↑Thrb2 (liver)*	*↑Tshr* *↔Trhr*		*↔Trh* *↑Tshb*
Atrazine (1232, 4311)										↔	
Boron (2736)	↑NIS									↔	
Butyl, ethylparaben (2015)										↔	
Carbendazim (1533)										Abnormal morphology	
Corn oil (*21808*, 3726*)				*↑1a1*	*↑1b1*					Abnormal morphology	
Curcumin (208, 16261, 3726*)										↔	
DE-83R (33)				↔ T4 UGT activity		↔EROD ↔PROD					
Atrazine metabolites (18277)										↔	
EE (*2015, 275*)				*↔1a1*	*↔1c1, 3, 1b1*					↔	
NaNO3 (159)	↔NIS	*↑Tpo*	*↑Mct8*						↔TSHr	Abnormal morphology	*↓Tg* *Duox?*
PCB28 (2211)				↔ T4 UGT activity							
Saisentong (64)				*↓* T4 UGT activity						↔	
Soybean oil/protein (18846, *682, 14344*)										↔	
Zinc (*27670, 21344, 1536*),										Abnormal morphology (electron microscopy)	↑ iodide uptake
3-(dibutylamino) phenol (214)										↔/Abnormal morphology	

Chemicals grouped according to T4 – TSH response patterns and additional data regarding different THS endpoints extracted from the articles. Patterns of hormonal changes are depicted as follows: decreased T4 and increased TSH (↓T4, ↑TSH), decreased T4 and unaltered TSH (↓T4, ↔TSH), unaltered T4 and TSH (↔T4, TSH). Cadima reference numbers in green refer to Tier 1 studies (Definitely or probably low risk of bias) and in black for Tier 3 studies (Definitely or probably high risk of bias). Cadima reference numbers in italics stand for the single-dose studies. THS endpoints are organised as follows: synthesis of TH (NIS and TPO), distributor proteins and TH transporters, liver enzyme induction (T4-UDPGT, other UGTs, SULTs and other markers), deiodinases (DIO), TH receptors (THr), TSH and TRH receptors (TSHr, TRHr), thyroid histology and other markers. When a chemical induced an increased response, this was marked with a “↑”; no effect “↔” and decreased response with a “↓”. Outcomes without a clear response are marked with “?”. Readouts are written in capital letters for enzymatic activity/protein levels, and in italics for gene expression. pit, pituitary; hyp, hypothalamus; liv, liver. Mice studies are shadowed in grey. Empty cells indicate that an endpoint was not assessed. Cadima reference number with asterisk (*) indicates that fT4 was measured instead of T4.

The majority of chemicals that produced T4 decrements with concomitant TSH increases also caused histopathological changes in the thyroid gland. Whilst it cannot be excluded that these findings are related to the normal higher variability of TSH levels and/or methodological issues around TSH measurements, we also extracted information for potential mechanistic explanation. In some cases, this was accompanied by an increase in iodide uptake (reported for polybrominated biphenyls (PBB), spirolactone or TCDD). In general, most test compounds interacted with multiple targets, such as DE-209, melamine or perchlorate. These were shown to affect TH synthesis, DIO activity and the central regulation via TSH receptor (TSHr). Several studies reported liver enzyme induction after chemical exposure; for example, doxylamine succinate, phenobarbital, spirolactone, TCDD, thiazopyr, thiodiazole copper and tributyltin. Two studies with pregnenolone and thiazopyr showed an increase in T4 biliary excretion. Expression of PAX8, *Pax8* and *Ttf1*, transcription factors involved in the regulation of thyroid-related genes, was altered after exposure to DE-209 and tributyltin.

Most treatments resulting in decreased serum T4 levels without concomitant TSH increase likely exhibit an extrathyroidal mechanism mediating their effect by liver induction; particularly, through liver microsomal enzyme induction, such as T4-UDPGT. Examples are Aroclor 1254, fluopyram, myclobutanil or triclosan. In addition, BROD, EROD, MROD or SULTs were frequently upregulated by several compounds, such as bromkal, DE-79 or PCB118. Similarly, Aroclor 1254 and 3-MC were able to increase the biliary excretion of T4, increasing T4 clearance in serum after Aroclor 1254 treatment but not for 3-MC. PFOS increased the loss of TH via urine along with decreased levels of TH in serum and liver. NIS/*Nis* and TPO/*Tpo* were assessed for several test compounds, most of them showing no change. Aroclor 1254, DEHP, fipronil or fluoropyram, among others, altered thyroid morphology. Other chemicals such as DE-47, flutamide, myclobutanil or TBBPA did not induce any detectable histological thyroid gland disruptions.

Limited data were available for studies reporting unchanged levels of T4 and TSH. Data retrieved from these studies did not show consistent results. For example, acrylamide or NaNO_3_ mediated their effect through targeting the gene expression of *Tpo*, *Dio3*, *Tshr*, *Tshb* or *Tg*, but without changes in TH/TSH. Further, the pesticide saisentong decreased UDPGT activity without notable changes in thyroid histomorphology. The solvent carrier corn-oil upregulated the genes coding for *Ugt1a1* and *Sult1b1* which was associated with abnormal thyroid morphology.

## 4 Discussion

Regulatory assessment of THSDCs currently relies on the observation of histopathological findings in the thyroid as an indicator for adversity in combination with altered T4/TSH levels, which on their own would be considered to be of concern (14, 23, 42, 43). A more comprehensive understanding of the mechanisms underlying THS disruption, together with refinement of existing tests and development of novel approaches, would increase the weight of evidence for chemical assessments. This, in turn, would further improve the interpretation of test data and strengthen confidence in the identification of THSDCs (14, 42, 44).

This study investigated whether THSDCs act on the TH-system by reducing serum T4 with a compensatory increase in TSH consistent with the canonical HPT-axis negative feedback model. We found that this pattern was not always observed, and some chemicals can disrupt TH homeostasis without exhibiting these specific changes in serum hormone concentrations. Therefore, restricting the identification of THSDCs to the expectation that they would induce a reduction in T4 together with increased TSH serum concentrations may not capture chemicals that produce alternative, toxicologically relevant response patterns. Here we present a comprehensive review of the peer-reviewed literature using systematic review methodology and RoB analysis. Our aim was to relate patterns of TH levels following chemical exposure to MoA information in support of test method development and to inform the testing strategy for the regulatory assessment of chemicals. We summarized our findings in a narrative synthesis as it was not possible to conduct a quantitative meta-analysis due to data heterogeneity in the selected studies.

Current European regulations focus on thyroid histopathology and changes in serum TH concentrations to evaluate *in vivo* adverse effects of chemicals. In addition, older datasets which were produced before the currently applicable European Chemicals Agency (ECHA)/EFSA guidance (23), frequently only provide information on histopathology. However, reliance on these parameters may not be sufficient to detect potential THSDCs which act through other mechanisms (45, 46). In fact, serum TH levels may not reflect tissue levels as autonomous regulation of TH exists at tissue level independent of the HPT-axis (7, 47–49). The interpretation of test results is often based on the canonical negative feedback loop of the HPT-axis (45, 48). In this “idealized view”, lowered T4 will lead to a chronic increase in TSH levels and ultimately, to thyroid morphological changes. However, this negative feedback loop is not always observed in experimental studies, meaning that chemicals that can adversely affect the thyroid by displaying different patterns, e.g. if only T4 is increased, or TSH changes in the absence of effects on T4 (**Tables 5**, **6**, **7**), may be disregarded without suitable tests for other thyroid relevant endpoints.

Additionally, as pointed out by Beekhuijzen and colleagues (50), the ECHA/EFSA guidance does not specify a threshold for TH changes which would identify a chemical as a THSDC. This was also a challenge in our analysis, as we found a broad range of percentage changes in studies reporting significant results, and conversely, similar ranges that were not significant in other studies (**Supplementary Materials 11–13**).

Scanning the literature using generic terms for EDCs and the THS retrieved over 25,000 records which were screened for relevance. Application of inclusion/exclusion criteria to the search results and duplicate removal identified 908 studies for full-text screening and, ultimately, the selection of 234 articles. The studies included here cover a wider range of chemicals compared to our previous systematic review for the gestational/lactational period (25). The selected studies reported treatments with chemicals which were roughly grouped into bisphenols and tetrabromobisphenol, phthalates, pesticides, personal care products, pharmaceuticals, polychlorinated-, polybrominated- and perfluorinated compounds, heavy metals, industrial chemicals, mix/dietary supplements. As highlighted in our previous study, a limited set of chemicals were examined during the perinatal period with some chemical groups underrepresented. The number of pharmaceuticals, personal care products or industrial chemicals are also limited (compounds grouped per chemical class in **Supplementary Materials 5, 6**).

Our RoB assessment highlighted deficiencies in TH/TSH quantification due to the lack of reported method details or appropriate adaptation of commercial kits for human THs to rodent experiments (2). In general, there is a lack of standardized assay methodologies, reference materials or harmonized assays used for rodent serum analyses of TH and TSH concentrations and there remains an urgent need for standardization (35, 50). This question in the RoB therefore generally scored as “definitely high” or “probably high” risk of bias. There is also a lack of reporting standards for rodent TH assessment methodology, including the materials and methods, data analysis, and standardized reference intervals. As previously discussed (45, 46), validated OECD test methods do not adequately address the identification of THS disruption and the adverse effects in the developing organism, nor in adults. Nevertheless, the few existing OECD guidelines remain instrumental for the identification of THSDCs (51). Most of the reviewed studies did not follow test guidelines or other standard methodology which ensures standardized reporting. Adherence to reporting standards in animal experiments and toxicology testing would help to improve the reliability and reproducibility of THSDC testing (45, 52–55). Recommendations to validate, standardize, and harmonize the methodology of TH and TSH analytics are currently under discussion (8, 35, 42, 43). In an effort to promote standardization, some recommendations for T4/TSH measurements are available in the ATA Guide (2).

Another methodological shortcoming was the use of soy-containing diets in rodent models, which may interfere with thyroid function (e.g. study #16718). For example, TPO activity was 50% lower in rats consuming a soy-based diet than in rats fed a soy-free diet. However, no impact on serum T4 and TSH, and thyroid histopathology was observed between untreated and treated groups (56). Another isoflavone, genistein, blocks T3 uptake via MCT8 and inhibits T4 binding to TTR or TBG (57, 58). Other targets of flavonoids are NIS, DIOs or TRb (59–62). Some authors hypothesized that other factors, such as iodine-deficient diets or chemical exposure, may induce hypothyroidism in soy-fed rats (7, 38).

ATA guidelines recommend measuring circulating levels of T4 in serum rather than plasma (2), as anticoagulants in the plasma may affect the quantification of free TH levels. While most studies quantified TH in serum, there were still some which measured plasma levels, which may confound the reliable detection of hormonal levels. It should also be noted that in humans, usually free TH levels are determined whereas the total serum TH are measured in rats. This is due to differences in TH protein-binding characteristics between humans and rodents and a limited blood volume (43).

Our evidence mapping revealed a mixed quality of inter-study data reproducibility for T4/TSH levels and divergent patterns were observed for some compounds which were tested in different independent studies. This can partly be explained by differences in dose ranges, treatment duration or timing of TH measurements after the last chemical administration. The better agreement for increases in T4 compared to the differences observed for TSH may also be due to differences in sensitivity and reproducibility of analytical methods for both hormones and the higher abundance of T4 compared to TSH (63). Sexual dimorphisms in the response to THSDC exposure may also explain some of the differences (2, 43). The divergent patterns observed, for example, with chlorpyrifos, amiodarone or di-n-butyl phthalate are harder to explain, as they all used similar doses and exposure duration. However, all these studies ranked as Tier 3, illustrating the urgent need to prioritise the standardisation and harmonization of experimental protocols and their reporting, and to implement new guidelines to avoid inconsistencies among studies.

Here we provide evidence that post-lactational exposures to certain chemicals disrupt the THS. Two main patterns became apparent: decreased T4 followed by an increase of TSH or by unaltered TSH. It should be noted that this systematic review was not intended to identify the full mechanistic explanations behind TH dysregulation as the THS has multiple targets for potential disruption and one chemical may target more than one TH endpoint, e.g. TH synthesis, transport, metabolism, action or regulation of the negative feedback of the HPT-axis (46, 64). Although we collected available mechanistic information from the included studies to elucidate potential MoAs, such data are usually scarce and sometimes contradictory (**Table 8**). Additionally, several other responses outside the canonical negative feedback loop were observed, such as increase or decrease of both T4 and TSH, or augmented T4 without TSH changes. It is also important to note the distinction between lowered T4 in clinical endocrinology versus toxicological testing. In humans, there are many clinically relevant conditions of changed TH serum concentrations not accompanied by expected TSH alterations. These could be due to mutations of various THS components or due to pathophysiological changes affecting the TH-axis and its feedback regulation, whereas toxicological tests are conducted in healthy animals in the absence of other conditions (65–67).

Our evidence mapping showed that chemicals falling into this first pattern (40 out of 184 units of evidence), the “idealized view” of the HPT-axis, included BPA, triclosan, TCDD or PTU, similar to our previous study for gestational exposures (25). Conversely, perchlorate is now included in this pattern, while DEHP is within the group of chemicals decreasing T4 without TSH changes. A common outcome in this group is abnormal thyroid gland morphology, however the capacity to interfere with TH synthesis is barely reported. NIS mediates active transport of perchlorate (68), albeit with variable stoichiometry because of perchlorate binding to a high-affinity non-transport allosteric site (69). Both perchlorate and DEPH upregulated *Nis* (**Table 8**), suggesting a compensatory response to declines in T4. In addition, TSH plays a major role in post-transcriptional regulation of NIS, upregulates NIS (and TPO) expression, and stimulates iodide uptake activity (70).

Next, our evidence mapping showed that 58 out of 184 units of evidence for multi-dose studies reported reductions in T4 without accompanying changes in TSH concentrations. The liver plays a key role in determining T4 blood levels via a complex network of processes involving transporters which move THs into cells, converting T4 to active or inactive forms via deiodinases, and clearing excess circulating hormones through sulfation and glucuronidation. It also synthesizes key TH-binding proteins and albumin, which maintain a balance between active and inactive THs in the bloodstream (24, 49). In addition, certain chemicals can activate xenobiotic-sensing transcription factors (e.g., CAR, PXR or AhR), which can increase peripheral elimination of T4 via activation of UGTs which catalyze glucuronidation of T4. T4 and T3 are differentially excreted as they are glucuronidated by different UGT isoforms (24). THSDCs can selectively inhibit the different isoforms of liver enzymes, via deiodinase inhibition or through selective inhibition of cytochrome P450 enzymes, thus altering the T3:T4 ratio in the serum and consequently, TSH levels. As reviewed in Vansell (24), induction of T3-glucuronidation, likely via PXR or CAR, instead of T4-glucuronidation may increase circulating TSH. The reduction in active T3 provides stronger negative feedback to the pituitary gland to suppress TSH compared to T4. Therefore, when T3 levels in circulation decrease, the pituitary is prompted to increase TSH production. In contrast, as T4 is a prohormone, adequate feedback can be maintained converting remaining T4 to T3, to keep TSH levels stable. However, Vansell did not rule out that both T3- and T4-glucuronidation and increased hepatic uptake and biliary export may be behind the increase in TSH (24).

The finding that low levels of T4 do not inevitably induce TSH was observed for Aroclor 1254 and some PCB congeners. Reduction of T4 by different PCBs is not always correlated with UGT activity or with increased biliary excretion of T4-glucuronide (71). These authors reported the rapid clearance of [^125^I]T4 from the plasma suggesting that these compounds might have greater dependency on the uptake of T4 by transport proteins (e.g. Oatp1a4). However, it is not clear why a reduction in T4 did not produce a TSH response. For example, Vansell and Klaasen (study #321) described increased T4 biliary excretion for 3 compounds (pregnenolone, 3-MC and Aroclor 1254) which are not consistent with changes in TSH. While the levels of TSH after pregnenolone treatment were increased, 3-MC and Aroclor 1254 did not induce such TSH increments. PCBs, which can mimic TH and bind to TRs, can potentially block TSH release (72, 73). Due to the mixed quality of reporting, and general methodological differences between T4 and TSH detection, it also cannot be ruled out that changes in TSH may not have been significant in some studies due to larger data variability, again illustrating the importance of assay standardization and reporting guidelines. Reporting of historical background control data would further support the data interpretation if TSH changes could not be detected.

Finally, a smaller set of chemicals showed no change in T4 or TSH levels, however they affected other endpoints, like abnormal thyroid morphology in very few studies, e.g., with carbendazim (damaged follicles, basophilic cytoplasm, lymphoid cell infiltration, enlargement interstitial tissue, study #1533), NaNO_3_ (increased colloids, thyrocyte atrophy, membrane rupture and lymphocyte infiltration, study #159), and 3-(dibutylamino)phenol (the highest concentration caused follicular epithelial cell hypertrophy in 1 male out of 10, study #214). For acrylamide, thyroidal *Tpo* and *Mct8* were upregulated at the highest concentration (study #13789), along with *Tshr*, and hepatic *Thra1* and *Thrb2*. A similar pattern was observed for the highest concentration of NaNO_3_ upregulating *Mct8* and *Tpo* (study #159).

Their MoA may involve activation or inhibition at peripheral levels where they could target nuclear receptors leading to changes in the expression of thyroid-related genes (74). Such compounds would currently escape detection as THSDCs as they do not change the hormone levels, although they are able to induce adverse effects on the THS.

Finally, proposals to improve the detection of THSDCs include the updating of existing *in vivo* test methods to include molecular endpoints, together with improvement of methods such as validated TH measurements, and standardized reporting (35, 44), as well as the testing of chemicals that raised concern in other tests in the comparative thyroid assay (CTA) (42, 75).

The implementation of an Integrated Approach to Testing and Assessment (IATA) for THSDCs could help to fill gaps in our understanding of THS disruption. Collating existing mechanistic data for suspected THSDCs as well as validation and acceptance of New Approach Methods (NAM) to generate appropriate data for integration in Adverse Outcome Pathways (AOP) would be a way forward for a better understanding of potential MoAs and clearer identification of THSDCs (42, 44, 46, 76, 77).

## 5 Conclusion

We conducted this systematic evidence mapping exercise to present a comprehensive overview of TH patterns observed after exposure to THSDCs in adult rodents. We have also collated MoA information when provided and related this to the observed patterns to better understand the impact of THSDCs and increase the knowledge base for improved regulatory testing.

Most compounds decreased the levels of serum/plasma T4, but different responses for TSH were observed. Thus, we conclude that the current “idealized view” of the disruption of HPT-axis does not apply to the majority of compounds assessed here. Therefore, it is crucial to not solely rely on the canonical HPT-axis hormonal patterns to determine the adversity of a chemical. Furthermore, most compounds included in this systematic map may interfere with the THS at multiple levels from TH synthesis to peripheral metabolism, however, the mechanisms by which they affect each step have not yet been fully identified. Debates are taking place regarding the adequacy of the current OECD test guidelines and EU testing requirements to detect THSDCs; currently they require changes in serum T4 concentrations (indicator for endocrine MoA) together with changes in thyroid gland histopathology (indicator for adversity). In contrast, serum T4 concentration changes alone are seen as sufficient to raise concern within clinical endocrinology, but not for chemical regulation. In this view, chemicals which do not lead to thyroid histopathological effects may still be capable of thyroid disruption but might escape identification as being potentially harmful to human health according to current regulatory requirements. Improvements in the standardization of TH analytical methods, together with transparent reporting of assay performance, test results, and other study characteristics, are needed to improve confidence in the interpretation of thyroid-related endpoints. In addition, existing *in vivo* test guidelines should be reviewed to determine whether THS related endpoints are adequately captured. Combined with a well-developed IATA, these improvements would facilitate a more reliable identification of THSDCs. Furthermore, changes in T4 observed *in vivo* may provide sufficient evidence of thyroid hormone system disruption when supported by mechanistic evidence and AOPs linking the observed endocrine activity to adverse outcomes.

## Data Availability

The original contributions presented in the study are included in the article/**Supplementary Material**. Further inquiries can be directed to the corresponding author.

## Glossary

3-MC: 3-methylcholanthrene
AhR: Aryl hydrocarbon receptor
AOP: Adverse Outcome Pathway
ATA: American Thyroid Association
BPA: Bisphenol A
BROD: 7-benzyloxyresorufin oxidation
CAR: Constitutive Androstane receptor
CTA: Comparative thyroid assay
CYP: Cytochrome P-450
DE-*x*: Polybrominated diphenyl ether-*x* (47, 71, 79, 83r, 99 or 209)
DEHP: Bis(2-ethylhexyl) phthalate
DIO: Deiodinases
DUOX: Dual oxidases
ECHA: European Chemicals Agency
EDC: endocrine-disrupting chemical
EFSA: European Food Safety Authority
EPA: Environment Protection Agency
EROD: 7-Ethoxyresorufin-O-deethylase
FT4: Free thyroxine
FT3: Free triiodothyronine
HPT: Hypothalamic–pituitary–thyroid-axis
IATA: Integrated Approaches to Testing and Assessment
MCT8: Monocarboxylate transporter 8
MoA: Mechanism of action
MROD: 7-methoxyresorufin O-demethylation
NAM: Novel Approach Method
NIS: Sodium/iodide symporter
NTP: National Toxicology Program
OATP: Organic anion transporting polypeptide
OECD: Organisation for Economic Co-operation and Development/
OHAT: Office of Health Assessment and Translation
PBDE: Polybrominated diphenyl ethers
PCB: Polychlorinated biphenyl
PECO: Population, Exposure, Comparator, Outcome
PND: Postnatal Day
PPAR: Peroxisome proliferator-activated receptor
PROD: 7-pentoxyresorufin O-dealkylase
PXR: Pregnane X receptor
RoB: Risk of bias
SULT: sulfotransferases
T3: Triiodothyronine
T4: Thyroxine
TBBPA: Tetrabromobisphenol
TG: Thyroglobulin
TH: Thyroid hormone
THRSP: Thyroid hormone responsive protein
THS: Thyroid hormone system
THSDC: Thyroid hormone system disrupting chemical
TPO: Thyroid peroxidase
THr: Thyroid hormone receptor
TRH: Thyrotropin-releasing hormone
TSH: Thyroid stimulating hormone
TSHr: Thyroid stimulating hormone receptor
TTR: Transthyretin
UDPGT: T4-UDP-glucuronosyltransferase
UGT: UDP-glucuronosyltransferases
